# Computational Identification of RNF114 nsSNPs with Potential Roles in Psoriasis and Immune Dysregulation

**DOI:** 10.3390/medsci13030194

**Published:** 2025-09-16

**Authors:** Ghalia Mahfod Aldoseri, Arwa Ibrahim Alwabran, Ghanem Mahfod Aldoseri, Mobarak Mahfod Aldoseri, Ebtihal Kamal

**Affiliations:** 1 College of Medicine, Prince Sattam bin Abdulaziz University, Al-Kharj 16278, Saudi Arabia; aldoserighalia@gmail.com (G.M.A.);; 2 Department of Basic Medical Sciences, College of Medicine, Prince Sattam bin Abdulaziz University, Al-Kharj 16278, Saudi Arabia

**Keywords:** RNF114, single-nucleotide polymorphisms, psoriasis, bioinformatics, structural modelling, pathway enrichment

## Abstract

Background: RNF114 gene encodes an E3 ubiquitin ligase involved in immune signaling and regulation of inflammation. Genetic variants, particularly nonsynonymous single-nucleotide polymorphisms (nsSNPs), may interfere with protein function and cause immune diseases such as psoriasis. Although significant, the structural and functional impact of RNF114 nsSNPs is not well understood. Methods: We used comprehensive bioinformatics analyses to predict the functional impact of RNF114 nsSNPs. Deleterious variants were predicted by SIFT, PolyPhen-2, PROVEAN, META-SNP, ESNP&GO, PANTHER, and Alpha-Missense. Protein stability was examined by I-Mutant2.0, and MUpro further contextualized variant effects. Structural modeling was performed by AlphaFold and visualized using UCSF ChimeraX 1.10.1. Additionally, we studied the Conservation using ConSurf and protein-protein interaction by STRING tools. Results: Among 252 available nsSNPs, three mutations—C49R (rs1600868749), R68C (rs745318334), and R68H (rs758000156)—were predicted to have a deleterious and destabilizing effects on the protein structure by all the tools. All three variants were located in extremely conserved residues and were predicted to significantly destabilize the protein structure. Structural modeling demonstrated disruptions in the RNF114 domain structure. STRING analysis revealed interactions of RNF114 with key immune regulators, and pathway enrichment pointed to roles in NF-κB signaling, ubiquitin-mediated proteolysis, and autoimmune disease pathways. Conclusions: In the current study, we predicted three novel, potentially pathogenic RNF114 variants with protein-destabilizing effect that could lead to immune dysregulation.

## 1. Introduction

Psoriasis (MIM #177900) is a complex, chronic inflammatory skin disorder with a strong genetic basis, affecting approximately 2% of the population in Europe and North America [1]. It is characterized by keratinocyte hyperplasia and dense immunocellular infiltration [1,2,3]. A key aspect of psoriasis, keratinocyte hyperplasia, involves an increase in cell numbers and the thickening of the epidermis, known as acanthosis [4]. This hyperplasia is primarily driven by IL-17A, TNF-α, and IL-22, which are produced by T-helper 17 cells [5].

Genetic factors are pivotal in the development of psoriasis, with over 60 loci influencing an individual’s susceptibility to this condition. These loci are integral to immune regulation and the function of the epidermal barrier [6]. The RING finger protein 114 (RNF114) gene, situated on chromosome 20q13.13, encodes a protein consisting of 228 amino acids. (https://www.genecards.org/cgi-bin/carddisp.pl?gene=RNF114&keywords=RNF114, accessed on 15 April 2025).

Recent studies have identified RNF114, a gene encoding a novel ubiquitin-binding protein (E3 ubiquitin ligase), as a significant contributor to psoriasis susceptibility [7,8]. E3 ubiquitin ligases are integral to essential cellular processes, including innate immunity, the regulation of inflammation, and protein degradation via ubiquitin-dependent pathways. This function is crucial for the degradation of proteins involved in immune signaling, thereby modulating the overall immune response [9]. Single-nucleotide polymorphisms (SNPs) represent variations among individuals at a single nucleotide position within the DNA sequence [9]. Nonsynonymous SNPs (nsSNPs) result in alterations of amino acid residues due to changes in the DNA sequence at a single nucleotide position (A, T, C, or G). This modification contributes to the functional diversity of the associated proteins [10]. The role of RNF114 has been elucidated in various studies, underscoring its potential involvement in disease susceptibility, particularly in autoimmune disorders [11]. A genome-wide association study identified rs495337, which is linked to increased expression of RNF114 in skin and immune cells, suggesting it may function as a regulatory variant influencing psoriasis risk [8]. Additionally, rare promoter variants (c.-64C4A, c.-41C4T, c.-66C4A, and c.-9A4C) have been discovered to significantly reduce RNF114 expression, thereby directly affecting gene regulation in psoriasis patients [12]. Understanding the function of nsSNPs in disease mechanisms is crucial for advancing personalized medicine. By identifying specific nsSNPs related to disease susceptibility, treatments can be customized for individuals based on their genetic makeup, thereby enhancing their therapeutic effectiveness [13,14].

Bioinformatics tools have recently become indispensable for predicting deleterious nsSNPs and their associations with different diseases [10,15]. Despite their potential significance, the impact of RNF114 nsSNPs on protein structure and function remains insufficiently explored, highlighting a notable scientific gap. To our knowledge, limited research has investigated the nsSNPs of RNF114 utilizing bioinformatics tools. This study aims to elucidate the structural and functional characteristics of the most pathogenic variants of the RNF114 gene. We conducted a comprehensive analysis of RNF114 nsSNPs using bioinformatics prediction tools to identify pathogenic and deleterious nsSNPs, thereby providing novel insights into their role in immune dysregulation and establishing a foundation for future functional and clinical investigations.

In this study, we explored the impact of nsSNPs in the RNF114 gene on protein structure and function, emphasizing the novelty of our approach by incorporating advanced computational analyses to evaluate how these genetic variations can significantly alter protein stability and activity. Unlike previous research that mainly concentrated on specific disease associations, our study highlights the potential role of the predicted nsSNPs in influencing the functional dynamics of RNF114, a protein involved in various biological processes, including the regulation of immune responses. By clarifying the structural consequences of these nsSNPs, we offer new insights into the molecular mechanisms underlying RNF114′s role in disease, paving the way for future therapeutic strategies targeting these genetic variants.

## 2. Methods and Materials

A summary of all the bioinformatics tools and servers used in this study, was summarized in Table 1.

### 2.1. Data Retrieval

The *RNF114* human gene was retrieved from the National Center for Biotechnology Information (NCBI) database (https://www.ncbi.nlm.nih.gov/; accessed on 2 May 2025). Moreover, information on the *RNF114* gene in terms of SNP, such as the SNP ID, was acquired from the NCBI dbSNP (accessed on 2 May 2025). The protein ID and sequence were obtained from the Swiss-Prot database, specifically UniProtKB, with accession number Q9Y508 (https://www.uniprot.org/uniprotkb/Q9Y508/entry, accessed on 2 May 2025).

### 2.2. Prediction of Deleterious Ns SNPs of RNF114 Gene

Sorting Intolerant from Tolerant (SIFT) (http://sift.bii.a-star.edu.sg/, accessed on 3 May 2025). It predicts changes in protein function as a result of amino acid substitutions. Using the NCBI database, nsSNPs were downloaded and uploaded to SIFT. A cutoff value of 0.05 was used, with values below this threshold identified as damaging (deleterious) and those above as benign (tolerated) [16].

PolyPhen-2 (http://genetics.bwh.harvard.edu/pph2/, accessed on 3 May 2025) is a prediction software that examines the likely effect of amino acid substitutions on human protein structure and function. It incorporates evolutionary conservation analysis through various sequence alignments and structural modeling of the protein. PolyPhen-2 predicts variants as benign, possibly damaging, or probably damaging. These groupings were based on the Position-Specific Independent Counts (PSIC) score, which ranged from 0 to 1. Values near zero are regarded as benign; a score greater than 0.5 is often interpreted as possibly damaging, whereas scores above 0.85 are classified as probably damaging [17].

PROVEAN (https://www.jcvi.org/research/provean/, accessed on 6 May 2025). Provean is a tool used to predict the degree of effect on a protein’s biological function if amino acid substitutions or insertions/deletions (indels) occur, using a cutoff value of −2.5. Scores of −2.5 or below predict a deleterious effect, whereas scores above this cutoff are considered neutral [18].

META-SNP (https://snps.biofold.org/meta-snp/, accessed on 13 May 2025) is a consensus strategy for the prediction of the pathogenicity of missense variants of human proteins. It gathers the results of separate programs—SIFT, SNAP, PANTHER and PhD-SNP—and harmonizes them by applying a trained statistical model that adds confidence to the predictions. Instead of relying on one strategy, META-SNP weighs the tools to decide on disease-associated or neutral variants; thus, more credible and trustworthy assessments are made compared to those of standalone predictors [19].

E-SNPs&GO tool (https://esnpsandgo.biocomp.unibo.it/, accessed on 15 May 2025) was employed to evaluate the pathogenic potential of nsSNPs. This method leverages protein sequence embeddings from developed and advanced language models (ProtTrans T5) in addition to Gene ontology annotations. A support vector machine (SVM) classifier is then used and applied to generate a probability score indicating whether a given variant is likely to be disease-associated or neutral [20].

PANTHER 19.0 (https://pantherdb.org, accessed on 13 May 2025) predicts the functional consequences of protein mutations based on the Position-Specific Evolutionary Preservation (PSEP) score. This score specifies evolutionary conservation over time, with values at 200 million years (my) likely to be benign. In contrast, values ranging from 200–450 may be damaging, while those exceeding 450 are considered damaging [21].

### 2.3. Prediction of Missense Variant Pathogenicity

Alpha Missense is an adaptation of AlphaFold, which is fine-tuned on human and primate variant population frequency databases to predict missense variant pathogenicity. It works by combining the structural context and evolutionary conservation. This model achieves state-of-the-art results across a wide range of genetic and experimental benchmarks without explicit training on such data [22].

### 2.4. Protein Stability Analysis of Predicted RNF114 nsSNPs

I-Mutant2.0 (https://folding.biofold.org/i-mutant/i-mutant2.0.html, accessed on 25 May 2025) is a support vector machine that determines whether the protein stability increases or decreases as a result of a point mutation. The software uses sequence and structural data to output the likelihood of an increase or decrease in protein stability with a reliability index and delta G (Change in Gibbs’ free energy). Negative delta G values indicate destabilizing mutations, suggesting possible functional loss [23].

MUpro (accessed on 25 May 2025), available at http://mupro.proteomics.ics.uci.edu, is a machine-based learning tool that predicts whether a single amino acid mutation will affect the stability of a protein. MUpro provides a classification of whether a mutation increases or decreases protein stability using structural or sequence-based features. A negative prediction score implies decreased stability, whereas a positive score implies increased stability or retention of stability. MUpro also interprets the results based on Gibbs free energy (∆∆G), with confidence scores between −1 and 11 [24].

### 2.5. Three-Dimensional Structure Prediction and Visualization

AlphaFold is a sophisticated artificial intelligence technology created by DeepMind for precise protein structure prediction. By utilizing deep learning, AlphaFold predicts the three-dimensional protein conformation from the amino acid sequence by modeling spatial restraints and evolutionary couplings. Structural models derived from such predictions yield useful information regarding protein folding, stability, and possible functional regions, especially in the absence of experimental crystallographic information that may not be present [25]. The protein structure was obtained using the AlphaFold model. The input was the UniProt sequences of the RNF114 protein.

UCSF ChimeraX is an advanced molecular visualization software that enables interactive exploration and accurate rendering of high-resolution biomolecular structures. Users can examine protein conformations, locate mutation sites, visualize protein-protein and protein-ligand interactions, and visualize structural data from experimental or predicted sources. UCSF ChimeraX is ideal for annotating the structural consequences of amino acid substitutions and producing publication-quality figures for structural studies [26].

### 2.6. Conservation Analysis and Surface Accessibility Prediction of RNF114

The ConSurf bioinformatics tool (https://consurf.tau.ac.il, accessed on 4 July 2025) was used to study the evolutionary conservation of nsSNP positions in the RNF114 protein sequence. We submitted the FASTA sequence of the RNF114 protein to the server and screened for exposed, buried, and highly conserved residues [27].

### 2.7. Identification of nsSNPs in RNF114 Protein Domains

We submitted the FASTA sequence of the RNF114 protein to the InterPro server (https://www.ebi.ac.uk/interpro, accessed on 20 August 2025). It predicts protein families and conserved domains, and then we manually pinpoint the positions of nsSNPs within these domains [28].

### 2.8. Prediction of Protein-Protein Interactions

STRING is an online resource for inferred and known protein-protein interactions. It has data from multiple sites as inferred evidence of interactions, including experimental techniques, computational methods, and public text mining [29]. We utilized the STRING database to visualize the RNF114-protein interaction using the protein-by-name module of STRING; the input was the RNF114 protein name, and the output was a graphical representation of protein interactions. A confidence score > 0.4 was set as the significance threshold, and the maximum number of interactors to be shown was no more than 50. Additionally, we used STRING to identify the gene ontology (GO) and enriched Kyoto Encyclopedia of Genes and Genomes (KEGG) pathways associated with RNF114-related proteins.

## 3. Results

### 3.1. Distribution of RNF114 Gene SNPs

**Figure 1:**
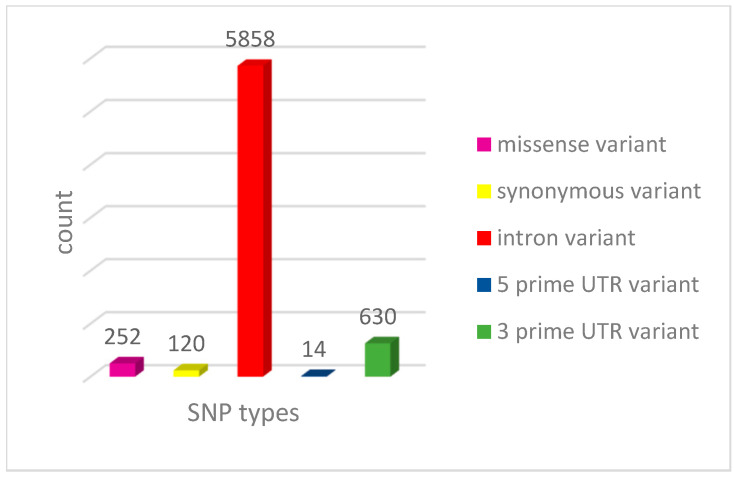
Distribution of SNPs in the RNF114 gene.

There were 252 nsSNPs, 120 were synonymous SNPs (sSNPs), and 5858 SNPs were in the intronic region. Meanwhile, 14 in the 5′UTR, and 630 in the 3′UTR (Figure 1). NsSNPs were selected for the investigation.

### 3.2. Detection of Functionally Damaging Missense Variants

We analysed the 252 nsSNPs for their potential deleterious effect. We first submitted the nsSNPs to SIFT and then those with deleterious effects only were submitted to PolyPhen2. Next, the nsSNPs that classified as probably and possibly damaging were further submitted to Provean and META-SNP tools (Table 2). Moreover, the nsSNPs that were predicted as disease causing by the first set of tools (SIFT, PolyPhen2, Provean, and META-SNPs), were further submitted to the second set of tools (ESNPs & GO, and PANTHER) (Table 3).

We further analyzed RNF114 nsSNPs using Alpha-Missense and found that all variants previously predicted as pathogenic by other computational tools were similarly classified as pathogenic in Alpha-Missense (Table 4).

### 3.3. Impact of nsSNPs on RNF114 Protein Stability

I-Mutant 2.0 and MUpro were used to predict the thermodynamic impact of amino acid substitutions based on changes in Gibbs free energy (ΔΔG). From this analysis, three missense mutations, C49R (rs1600868749), R68C (rs745318334), and R68H (rs758000156), were consistently predicted to destabilize the RNF114 protein. Interestingly, all three substitutions yielded negative ΔΔG values on both methods (Table 5).

### 3.4. Three-Dimensional Structure Prediction by AlphaFold and Visualization of Wild and Mutant Type Proteins by ChimeraX

**Figure 2:**
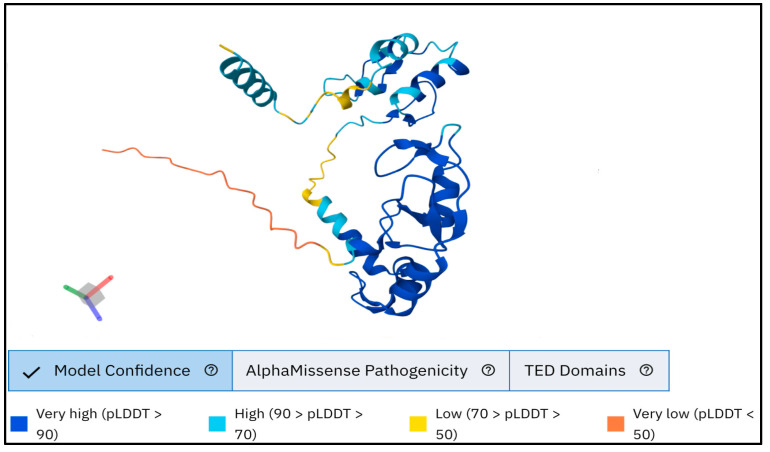
Protein 3D structure of human RNF114 predicted by AlphaFold2.

The AlphaFold prediction method provides a per-residue confidence value, pLDDT, between 0 and 100, which estimates the precision of every atomic position prediction. Residues with high pLDDT values (typically > 90) are predicted confidently and often fall in well-ordered regions, such as alpha-helical regions. In contrast, regions of medium (50 < pLDDT < 70) or low confidence (pLDDT < 50) are typically found to be flexible or open loops and consequently lack structure in isolation (Figure 2), where the structured domains are determined, and the low-confidence regions are represented accordingly.

**Figure 3:**
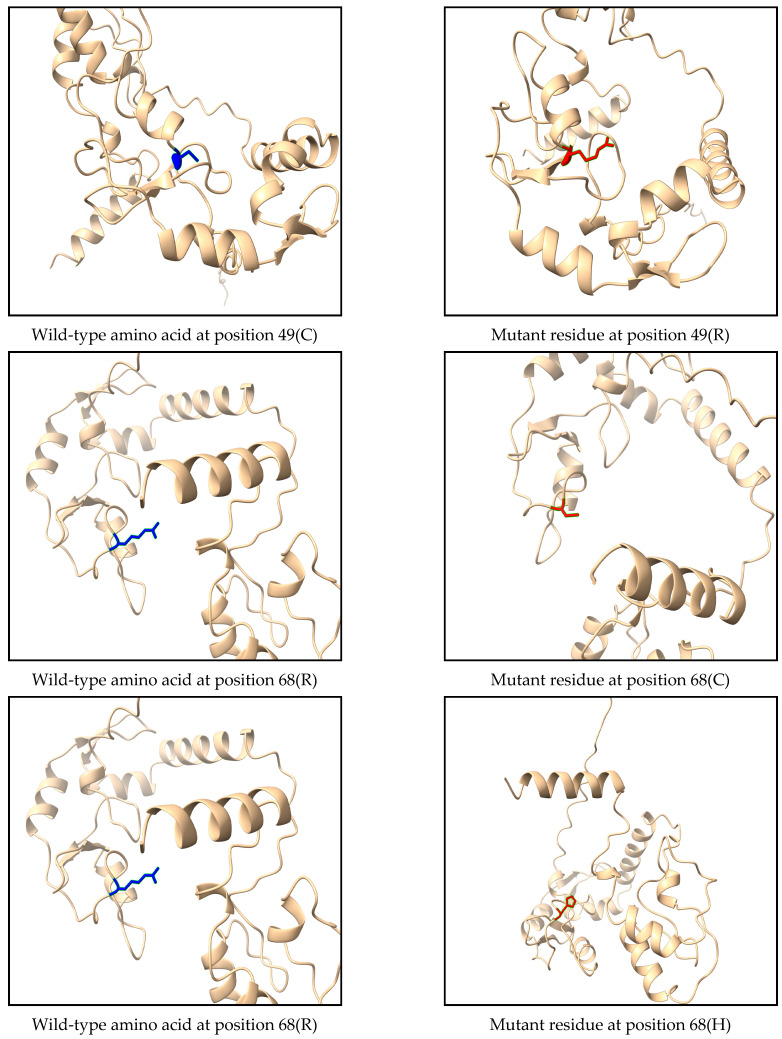
Effect of the three most deleterious nsSNPs on the RNF114 protein structure. ChimeraX was used to visualize the 3D structure of the wild type (blue), mutant residues (red).

ChimeraX software was employed to observe the 3D structure of the RNF114 protein, comparing visually the structural atoms of the wild-type amino acid (blue) and the mutated residues (red) as illustrated in Figure 3.

### 3.5. Evaluation of Conservation and Predicted Surface Accessibility for RNF114 Variants

Highly conserved residues are likely to be critical for the structure and function of a protein. We evaluated the conservation scores for the prioritized RNF114 variants, and all three (C49R, R68C, and R68H) received the highest conservation score of 9. R68C and R68H are predicted to be functional residues (highly conserved and exposed) that mediate interactions or regulation. In contrast, C49R is a structural residue (well conserved and buried), indicating that it can stabilize the core structure of the protein (Table 6). These characteristics support the hypothesis that these nsSNPs strongly disrupt RNF114 function and stability.

### 3.6. Domain Identification of the RNF114 Protein by the InterPro Server

The InterPro tool predicted the domain regions of the RNF114 protein. Zinc/RING finger domain, C3HC4 (zinc finger) at position (17–118), Drought induced 19 protein (Di19), zinc-binding at position (140–200), RING/U-box at position (23–70). RING-HC_RNF114 at position (26–71), Zinc finger RING-type profile at position (29–68), ring_2 at position (29–67), RING-type zinc-finger at position (29–64), Zinc finger C2HC RNF-type profile at position (91–110), C2HC Zing finger domain at position (85–117), and Drought induced 19 protein (Di19), zinc-binding at position (140–200) are as in Table 7.

### 3.7. RNF114-Interacting Proteins and Gene Ontology Analysis

**Figure 4:**
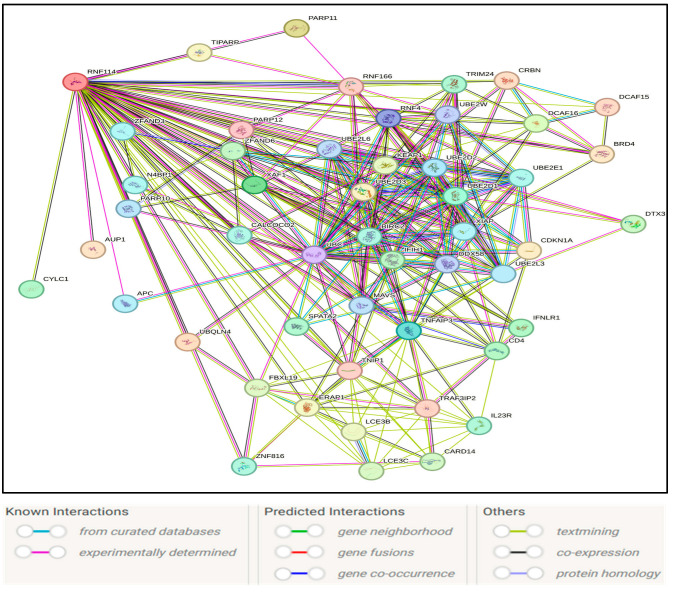
RNF114 protein interactions by STRING database.

Functional interaction mapping using the STRING database identified RNF114 as a hub in a densely connected protein-protein interaction network (Figure 4). RNF114 is directly linked to several key regulators of ubiquitination, apoptosis, and protein degradation pathways, including UBE2D1, UBE2D2, UBE2D3, RNF4, UBQLN4, TNFAIP3, PARP1, and PARP11. The adapter proteins XAF1 and DCAF16 were also found in the network, suggesting RNF114’s involvement in protein turnover and stress responses.

**Figure 5:**
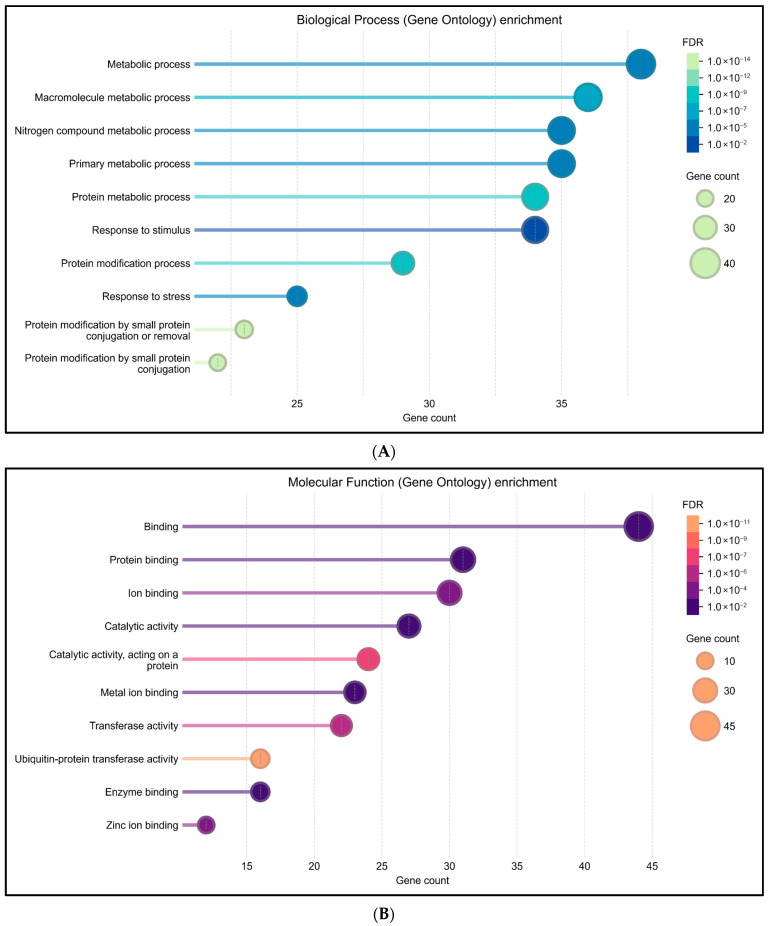

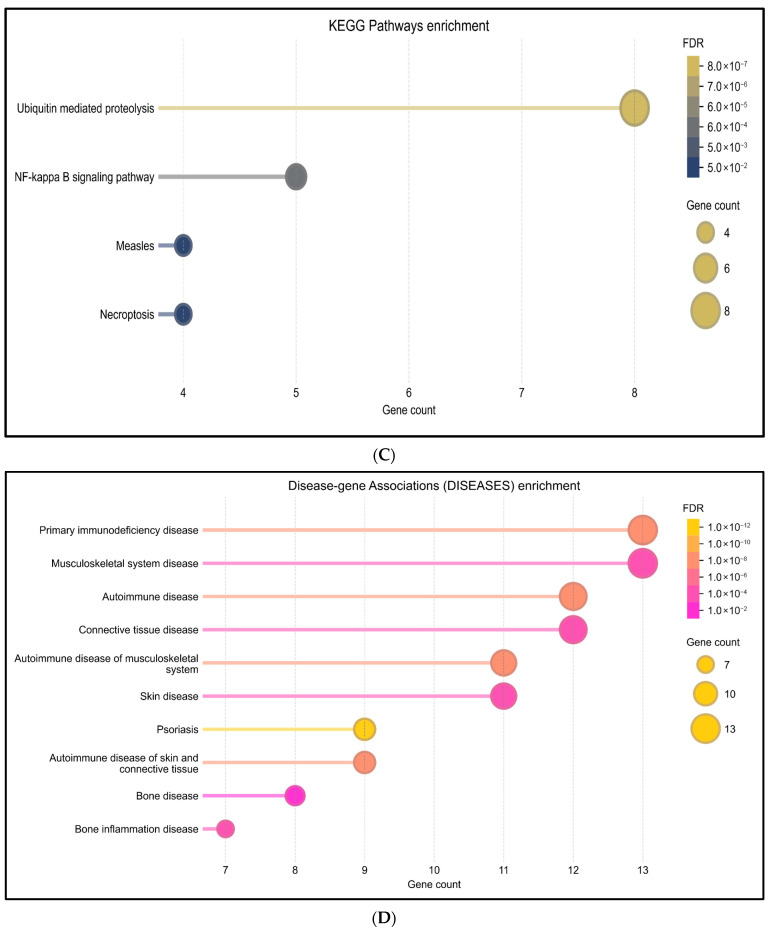
(**A**) Gene Ontology (Biological Process) enrichment, (**B**) Gene Ontology (Molecular function) enrichment, (**C**) KEGG pathway enrichment, (**D**) Disease-gene Associations enrichment analysis of RNF114-interacting proteins identified using the STRING database.

GO enrichment analysis of RNF114-interacting genes revealed significant overrepresentation in the most crucial biological processes, molecular functions, pathways, and disease associations, indicating its pervasive functional relevance. The biological process terms were heavily enriched for metabolic processes, macromolecule and nitrogen compound metabolism, response to stimulus and stress, protein modification processes, and small protein conjugation by ubiquitin (Figure 5A). Molecular function enrichment highlighted binding activity, catalytic and transferase activity, and ubiquitin-protein transferase activity (Figure 5B).KEGG pathway analysis also highlighted significant involvement in the pathways of ubiquitin-mediated proteolysis, NF-kappaB signaling, necroptosis, and viral infection, all of which involve cellular stress responses, and inflammatory signaling (Figure 5C). Disease-gene enrichment association connected RNF114-interacting genes with psoriasis, skin disease, autoimmune and connective tissue disorders, primary immunodeficiency, musculoskeletal system disorders, bone disease, and inflammation (Figure 5D). Together, these results indicate that RNF114 is a critical node in ubiquitin-related signaling pathways connected to immune regulation, proteostasis, and several immune-mediated and degenerative illnesses.

## 4. Discussion

We conducted a comprehensive bioinformatics analysis to evaluate the potential pathogenicity of non-synonymous single-nucleotide polymorphisms in the RNF114 gene, as well as their impact on the structure and function of the RNF114 protein. Initially, 252 nsSNPs were assessed using seven functional prediction programs: SIFT, PolyPhen, PROVEAN, META-SNP, SNP&GO, and Panther, to predict their potential pathogenicity (Table 2 and Table 3). From this screening, four candidate variants, specifically H46Y (rs146221262), C49R (rs1600868749), R68C (rs745318334), and R68H (rs758000156), were consistently predicted by all tools to be damaging and pathogenic.

Furthermore, Alpha Missense predicted that all three prioritized variants are likely to be pathogenic, as indicated by their high pathogenicity scores (C49R = 0.998, R68C = 0.947, R68H = 0.887) (Table 4). The agreement of these predictions with independent methods boosts confidence in suggesting these SNPs as candidates for future studies. Moreover, the Alpha-Missense classification is consistent with other predictive models, further supporting the identification of C49R (rs1600868749), R68C (rs745318334), and R68H (rs758000156) as potentially harmful variants.

Subsequently, we evaluated the impact of the predicted deleterious nsSNPs on the structural stability of the RNF114 protein using I-Mutant2.0 and MUpro. Both computational tools indicated a destabilizing effect for the variants C49R (rs1600868749), R68C (rs745318334), and R68H (rs758000156), as demonstrated by negative ΔΔG values indicative of conformational destabilization (Table 5). These findings suggest that such amino acid substitutions may disrupt proper protein folding, diminish domain integrity, and impair the E3 ubiquitin ligase function of RNF114.

Next, we studied the conservation status of three deleterious and destabilizing nsSNPs. They exhibited the highest conservation level of 9, reflecting significant evolutionary constraint. C49R (rs1600868749) was predicted as a structural residue characterized by being highly conserved and buried, suggesting its crucial role in maintaining the protein’s core stability. Meanwhile, R68C (rs745318334) and R68H (rs758000156) were categorized as functional residues, being highly conserved and accessible (Table 6), which implies they might be essential for facilitating interactions or regulatory functions. The three variants C49R (rs1600868749), R68C (rs745318334), and R68H (rs758000156), are located in the C3HC4 domain of RNF114 (Table 7), which is essential for RNF114 function as an E3 ligase by facilitating protein-protein interaction with an E2 enzyme [30,31].

STRING-based enrichment and protein-protein interaction analysis further elucidated the functional context of RNF114 (Figure 4). RNF114 is known to interact with various proteins associated with the ubiquitination pathway, including E2 conjugating enzymes and negative regulators of NF-κB signaling [32]. Gene Ontology analysis identified biological processes such as metabolic processes, protein modification processes, and responses to stimuli and stress. Molecular functions showed significant enrichment for binding, protein binding, and ion binding (Figure 5B). KEGG pathway enrichment analysis for RNF114 and its associated proteins indicated involvement in ubiquitin-mediated proteolysis and NF-κB signaling (Figure 5C). The findings align with the established role of RNF114 as an E3 ubiquitin ligase that interacts with A20/TNFAIP3 to negatively regulate NF-κB signaling, thereby mitigating pro-inflammatory responses [33,34]. The results of disease enrichment analyses further underscore this functional relationship by identifying associations with primary immunodeficiency disease, musculoskeletal and skin diseases, including psoriasis (Figure 5D). This is consistent with genetic evidence from genome-wide association studies that implicate RNF114 as a risk locus for psoriasis, where the gene is overexpressed in lesional tissue and contributes to dysregulated immune signaling [8]. Collectively, these enrichment analyses support the hypothesis that destabilizing missense variants in RNF114 may impair its function as a regulator of immune signaling and protein homeostasis, potentially leading to inflammatory diseases.

Interestingly, all three predicted nsSNPs (rs1600868749, rs745318334, and rs758000156) were not reported in Clinvar, nor have been associated with any specific disease in published literature (https://www.ncbi.nlm.nih.gov/clinvar/?term=rs1600868749, accessed on 20 August 2025), (https://www.ncbi.nlm.nih.gov/clinvar/?term=rs745318334, accessed on 20 August 2025), and (https://www.ncbi.nlm.nih.gov/) respectively. Moreover, none of these three nsSNPs have been reported in dbGAP GEO or OMIM. Their absence from the clinical databases suggests that these are novel deleterious variants with potential functional implications for the stability and function of RNF114, warranting further experimental investigation.

Our bioinformatics analyses played a crucial role in the initial prioritization of nsSNPs. To confirm the effects of these variants and determine their roles in disease processes, experimental evidence such as mutagenesis, protein stability assays, ubiquitination activity tests, and cellular signaling studies is essential. However, it should be noted that these findings are based solely on in silico predictions. While computation is beneficial for mechanistic hypotheses and prioritization, it cannot demonstrate functional effects in vivo. Experimental confirmation by site-directed mutagenesis, protein stability assays, ubiquitination activity assays, and cellular immune signaling assays should also form part of future studies. These experiments will be necessary to definitively confirm the pathogenic capability of these RNF114 variants and their involvement in diseases.

## 5. Conclusions

This study used comprehensive in silico tools to identify pathogenic nsSNPs in the immune signaling and ubiquitination-related gene, RNF114. By combining functional and stability predictions, structural modeling, conservation, and pathway analysis, we prioritized three variants, rs1600868749, rs745318334, and rs758000156, which showed strong structural destabilization and functional disablement. These variants reside in highly conserved positions, most likely disrupting RNF114 enzymatic activity and interactions and causing immune and inflammatory dysregulation.

Although these variants have not yet been annotated for clinical data in databases such as ClinVar, their pathogenic predictions strongly indicate their potential as key candidates for experimental validation. This validation could confirm their involvement in psoriasis and other immune disorders, thereby establishing them as valuable targets for drug development and diagnostic applications.

## 6. Recommendation

In this study, we explored the effects of nsSNPs while recognizing the significance of intronic SNPs, which, despite their crucial role in splicing reactions, are often overlooked or undervalued. Consequently, we recommend further comprehensive research into the functional implications of intronic SNPs.

## Figures and Tables

**Table 1:** Summary of the bioinformatics tools and servers.

Bioinformatics Tool/Server	Function	Input	Cut-Off Value
SIFT	Predicts whether an amino acid substitution affects protein function based on sequence homology.	SNP ID	Tolerance Index (TI) < 0.05 indicates deleterious. ≥0.05 indicates tolerated
PolyPhen-2	Assesses the impact of amino acid substitutions on protein structure and function using structural and evolutionary data.	Amino acid substitution	score ≥ 0.85 indicates probably damaging score > 0.5 < 0.85 possibly damaging <0.5 benign
PROVEAN	Evaluates the effect of amino acid substitutions on protein function by comparing sequences	Amino acid substitution	Score ≤ −2.5 indicates deleterious >−2.5 indicates benign
META-SNP	Predicts the pathogenicity of SNPs by integrating multiple algorithms (SIFT, SNAP, and PhD-SNP) for consensus.	Amino acid substitution	A consensus score >0.5 indicates pathogenic
E-SNPs&GO	Integrates support vector machine with functional annotations derived from Gene Ontology terms.	Protein sequence in the FASTA format and the list of variations	RI closer to 0 indicating benign variants, RI closer to 10 indicating pathogenic variants
PANTHER	Classifies nsSNPs based on evolutionary relationships and functional annotations.	Protein Sequence, and amino acid acid substitution	probably damaging (PSEP time > 450 my, possibly damaging(PSEP 450 my > time > 200 my, and probably benign (PSEP time < 200 my).
Alpha Missense	Predicts the impact of missense mutations on protein function using structural modelling.	UniProt ID, And official gene symbol	Pathogenicity score: 0–0.34: likely benign, 0.34–0.564: ambiguous, 0.564–0.78: likely pathogenic 0.78–1.0: likely pathogenic
I-Mutant2.0	Predicts changes in protein stability upon mutation by calculating ΔΔG values.	Amino acid sequence and Amino acid substitution	ΔΔG > 0 indicates decreased stability
MUpro	Predicts the effect of mutations on protein stability using machine-learning algorithms.	Amino acid sequence and Amino acid substitution	Score < 0.0 indicates destabilizing effect Score > 0.0 indicates stabilizing effect.
AlphaFold	Provides highly accurate protein structure predictions based on amino acid sequences	UniProt ID, official gene symbol	pLDDT > 90 Very high confidence 90 > pLDDT > 70 High confidence 70 > pLDDT > 50 Low confidence pLDDT < 50 Very low confidence
ChimeraX	Visualizes molecular structures and interactions, allowing for detailed analysis of protein conformations and mutations.	Protein structure data	N/A
ConSurf	Analyzes evolutionary conservation of amino acids to determine their functional importance.	protein sequence	Conservation score 9 indicates the highest conservation
InterPro	Integrates diverse protein family, domain, and functional site information to provide comprehensive functional annotations.	Protein sequence	N/A
STRING	Predicts protein-protein interactions to provide biological context for nsSNPs.	Protein name	Interaction score > 0.4 indicates significant interaction

∆∆G (changes in Gibbs free energy); PSEP (position-specific evolutionary preservation); pLDDT (a per-residue measure of local confidence).

**Table 2:** List of nsSNPs predicted to have deleterious effect by SIFT, PolyPhen2, Provean, and META-SNP.

	nsSNP ID	Amino Acid Changes	SIFT		PolyPhen2.0		Provean		META-SNP	
			Prediction	T1	Effect	Score	Effect	Score	Prediction	Score
1	rs2090286290	F27S	deleterious	0	possibly damaging	0.843	Deleterious	−4.344	Disease	0.71
2	rs1386412786	C29R	deleterious	0	probably damaging	1	Deleterious	−9.438	Disease	0.858
3	rs773986175	V40A	deleterious	0.03	possibly damaging	0.765	Deleterious	−2.68	Disease	0.675
4	rs1462212625	H46Y	deleterious	0	probably damaging	0.958	Deleterious	−4.792	Disease	0.791
5	rs1600868749	C49R	deleterious	0	probably damaging	1	Deleterious	−11.048	Disease	0.827
6	rs1305658652	C52R	deleterious	0	probably damaging	1	Deleterious	−10.453	Disease	0.772
7	rs745318334	R68C	deleterious	0	probably damaging	0.916	Deleterious	−5.546	Disease	0.793
8	rs758000156	R68H	deleterious	0	probably damaging	0.993	Deleterious	−3.848	Disease	0.773
9	rs772338253	S101C	deleterious	0.04	possibly damaging	0.881	Deleterious	−2.605	Disease	0.648
10	rs2090326008	S101P	deleterious	0.02	possibly damaging	0.799	Deleterious	−3.274	Disease	0.715
11	rs775686162	R104Q	deleterious	0.03	possibly damaging	0.67	Deleterious	−2.649	Disease	0.734
12	rs1017816166	R104W	deleterious	0.02	probably damaging	0.997	Deleterious	−6.345	Disease	0.787
13	rs2090326107	H106N	deleterious	0.02	probably damaging	0.965	Deleterious	−6.284	Disease	0.785
14	rs751133548	C110S	deleterious	0	probably damaging	0.998	Deleterious	−8.968	Disease	0.792
15	rs759089769	K112T	deleterious	0.02	possibly damaging	0.782	Deleterious	−4.729	Disease	0.569
16	rs775076155	R138C	deleterious	0	probably damaging	0.992	Deleterious	−5.423	Disease	0.736
17	rs2090330323	C143R	deleterious	0	probably damaging	0.999	Deleterious	−10.948	Disease	0.766
18	rs2090330344	P144S	deleterious	0	probably damaging	0.999	Deleterious	−6.444	Disease	0.721
19	rs1568920426	N150K	deleterious	0	probably damaging	0.992	Deleterious	−4.835	Disease	0.505
20	rs866758335	P174L	deleterious	0.02	probably damaging	0.999	Deleterious	−8.527	Disease	0.577
21	rs1443484485	P174T	deleterious	0	probably damaging	0.999	Deleterious	−6.91	Disease	0.576
22	rs766819838	C176R	deleterious	0	probably damaging	1	Deleterious	−11.035	Disease	0.755
23	rs754977174	M179T	deleterious	0	possibly damaging	0.834	Deleterious	−4.604	Disease	0.599
24	rs2090346648	P184S	deleterious	0.02	probably damaging	0.985	Deleterious	−5.709	Disease	0.501
25	rs772186536	H194R	deleterious	0	probably damaging	0.967	Deleterious	−7.013	Disease	0.67
26	rs866406478	R198Q	deleterious	0	probably damaging	0.978	Deleterious	−2.902	Disease	0.671
27	rs776148672	R198W	deleterious	0.04	probably damaging	0.998	Deleterious	−5.858	Disease	0.722
28	rs761458275	R200W	deleterious	0	possibly damaging	0.631	Deleterious	−3.729	Disease	0.696
29	rs2090346937	T205A	deleterious	0	probably damaging	0.998	Deleterious	−3.802	Disease	0.524
30	rs1262555907	T205I	deleterious	0	probably damaging	0.999	Deleterious	4.809	Disease	0.673
31	rs1160221496	F206S	deleterious	0	probably damaging	0.978	Deleterious	−5.9	Disease	0.755
32	rs2090357290	D212G	deleterious	0.01	probably damaging	0.998	Deleterious	−5.718	Disease	0.686

**Table 3:** List of pathological nsSNPs predicted by ESNPs & GO, and PANTHER.

	NsSNP	Amino Acid Change	ESNP & GO		Panther	
			Prediction	RI	Effect	PSEP
1	rs146221262	H46Y	Pathogenic	7	Probably damaging	1628
2	rs1600868749	C49R	Pathogenic	8	Probably damaging	1628
3	rs745318334	R68C	Pathogenic	7	Probably damaging	910
4	rs758000156	R68H	Pathogenic	4	Probably damaging	910

PSEP (position-specific evolutionary preservation).

**Table 4:** Alpha-Missense prediction of the pathogenic nsSNPs in RNF114.

	NsSNP	Amino Acid Change	Alpha-Missense Pathogenicity	Alpha-Missense Prediction
1	rs1600868749	C49R	0.998	Likely Pathogenic
2	rs745318334	R68C	0.947	Likely Pathogenic
3	rs758000156	R68H	0.887	Likely Pathogenic

**Table 5:** Deleterious and pathogenic nsSNPs predicted to have a significant decrease in protein stability by I-MUTANT 2.0 algorithm, and MUpro.

	NsSNP	Amino Acid Change	I-Mutant 2.0		Mupro		
			Stability	RI	DDG (kcal/mol)	Stability	DDG (kcal/mol)
1	rs1600868749	C49R	Decrease	5	−0.84	Decrease	−0.20914
2	rs745318334	R68C	Decrease	5	−0.27	Decrease	−0.22222442
3	rs758000156	R68H	Decrease	8	−0.44	Decrease	−0.72417906

**Table 6:** Conservation profile of the most damaging nsSNPs of RNF114.

	NsSNP	Amino Acid Change	Conservation Score	Prediction
1	rs1600868749	C49R	9	structural residue (highly conserved and buried)
2	rs745318334	R68C	9	functional residue (highly conserved and exposed)
3	rs758000156	R68H	9	functional residue (highly conserved and exposed)

**Table 7:** Domain regions of the selected most damaging nsSNPs in RNF114.

RNF114 Protein Domains	Positions	nsSNPs
Zinc/RING finger domain, C3HC4	17–118	C49R, R68C, R68H
Drought induced 19 protein (Di19), zinc-binding at position	140–200	
RING/U-box	23–70	C49R, R68C, R68H
RING-HC_RNF114	26–71	C49R, R68C
Zinc finger RING-type profile	29–68	C49R, R68C, R68H
ring_2	29–67	C49R
RING-type zinc-finger	29–64	C49R
Zinc finger C2HC RNF-type profile at position	91–110	
C2HC Zing finger domain	85–117	
Drought induced 19 protein (Di19), zinc-binding	140–200	

## Data Availability

The original contributions presented in this study are included in the article. Further inquiries can be directed to the corresponding author.

## References

[1] Ippagunta S.K., Gangwar R., Finkelstein D., Vogel P., Pelletier S., Gingras S., Redecke V., Häcker H. (2016). Keratinocytes contribute intrinsically to psoriasis upon loss of *Tnip1* function. Proc. Natl. Acad. Sci. USA.

[2] Ni X., Lai Y. (2020). Keratinocyte: A trigger or an executor of psoriasis?. J. Leukoc. Biol..

[3] Yang L., Fan X., Wang G. (2017). 016 Hyperactivation of Nrf2 contributes to keratinocyte hyperplasia in psoriasis by promoting Keratin 6, 16 and 17 expressions. J. Investig. Dermatol..

[4] Niehues H., Rikken G., van Vlijmen-Willems I.M.J.J., Rodijk-Olthuis D., van Erp P.E.J., Zeeuwen P.L.J.M., Schalkwijk J., van den Bogaard E.H. (2022). Identification of keratinocyte mitogens: Implications for hyperproliferation in psoriasis and atopic dermatitis. JID Innov..

[5] Lowes M.A., Suárez-Fariñas M., Krueger J.G. (2014). Immunology of Psoriasis. Annu. Rev. Immunol..

[6] Tsoi L.C., Stuart P.E., Tian C., Gudjonsson J.E., Das S., Zawistowski M., Ellinghaus E., Barker J.N., Chandran V., Dand N. (2017). Large scale meta-analysis characterizes genetic architecture for common psoriasis associated variants. Nat. Commun..

[7] Bijlmakers M.-J., Kanneganti S.K., Barker J.N., Trembath R.C., Capon F. (2011). Functional analysis of the RNF114 psoriasis susceptibility gene implicates innate immune responses to double-stranded RNA in disease pathogenesis. Hum. Mol. Genet..

[8] Capon F., Bijlmakers M.-J., Wolf N., Quaranta M., Huffmeier U., Allen M., Timms K., Abkevich V., Gutin A., Smith R. (2008). Identification of ZNF313/RNF114 as a novel psoriasis susceptibility gene. Hum. Mol. Genet..

[9] Kamal E. (2025). In Silico Prioritization of STAT1 3′ UTR SNPs Identifies rs190542524 as a miRNA-Linked Variant with Potential Oncogenic Impact. Non-Coding RNA.

[10] Kamal E., Kaddam L.A., Ahmed M., Alabdulkarim A. (2025). Integrating Artificial Intelligence and Bioinformatics Methods to Identify Disruptive STAT1 Variants Impacting Protein Stability and Function. Genes.

[11] Armstrong D.L., Zidovetzki R., E Alarcón-Riquelme M., Tsao B.P., A Criswell L., Kimberly R.P., Harley J.B., Sivils K.L., Vyse T.J., Gaffney P.M. (2014). GWAS identifies novel SLE susceptibility genes and explains the association of the HLA region. Genes Immun..

[12] Onoufriadis A., Simpson M.A., Burden A.D., Barker J.N., Trembath R.C., Capon F. (2012). Identification of Rare, Disease-Associated Variants in the Promoter Region of the RNF114 Psoriasis Susceptibility Gene. J. Investig. Dermatol..

[13] Momozawa Y., Mizukami K. (2021). Unique roles of rare variants in the genetics of complex diseases in humans. J. Hum. Genet..

[14] Folkersen L., Hooft F.V., Chernogubova E., Agardh H.E., Hansson G.K., Hedin U., Liska J., Syvänen A.-C., Paulsson-Berne G., Franco-Cereceda A. (2010). Association of Genetic Risk Variants with Expression of Proximal Genes Identifies Novel Susceptibility Genes for Cardiovascular Disease. Circ. Cardiovasc. Genet..

[15] Clifford R.J., Edmonson M.N., Nguyen C., Scherpbier T., Hu Y., Buetow K.H. (2004). Bioinformatics Tools for Single Nucleotide Polymorphism Discovery and Analysis. Ann. N. Y. Acad. Sci..

[16] Kumar P., Henikoff S., Ng P.C. (2009). Predicting the effects of coding non-synonymous variants on protein function using the SIFT algorithm. Nat. Protoc..

[17] Adzhubei I., Jordan D.M., Sunyaev S.R. (2013). Predicting functional effect of human missense mutations using PolyPhen-2. Curr. Protoc. Hum. Genet..

[18] Choi Y., Chan A.P. (2015). PROVEAN web server: A tool to predict the functional effect of amino acid substitutions and indels. Bioinformatics.

[19] Capriotti E., Altman R.B., Bromberg Y. (2013). Collective judgment predicts disease-associated single nucleotide variants. BMC Genom..

[20] Manfredi M., Savojardo C., Martelli P.L., Casadio R. (2022). E-SNPs&GO: Embedding of protein sequence and function improves the annotation of human pathogenic variants. Bioinformatics.

[21] Mi H., Muruganujan A., Thomas P.D. (2013). PANTHER in 2013: Modeling the evolution of gene function, and other gene attributes, in the context of phylogenetic trees. Nucleic Acids Res..

[22] Cheng J., Novati G., Pan J., Bycroft C., Žemgulytė A., Applebaum T., Pritzel A., Wong L.H., Zielinski M., Sargeant T. (2023). Accurate proteome-wide missense variant effect prediction with AlphaMissense. Science.

[23] Capriotti E., Fariselli P., Calabrese R., Casadio R. (2005). Predicting protein stability changes from sequences using support vector machines. Bioinformatics.

[24] Cheng J., Randall A., Baldi P. (2006). Prediction of protein stability changes for single-site mutations using support vector machines. Proteins Struct. Funct. Bioinform..

[25] Varadi M., Anyango S., Deshpande M., Nair S., Natassia C., Yordanova G., Yuan D., Stroe O., Wood G., Laydon A. (2022). AlphaFold Protein Structure Database: Massively expanding the structural coverage of protein-sequence space with high-accuracy models. Nucleic Acids Res..

[26] Meng E.C., Goddard T.D., Pettersen E.F., Couch G.S., Pearson Z.J., Morris J.H., Ferrin T.E. (2023). UCSF ChimeraX: Tools for structure building and analysis. Protein Sci..

[27] Ashkenazy H., Erez E., Martz E., Pupko T., Ben-Tal N. (2010). ConSurf 2010: Calculating evolutionary conservation in sequence and structure of proteins and nucleic acids. Nucleic Acids Res..

[28] Mulder N.J., Apweiler R., Attwood T.K., Bairoch A., Bateman A., Binns D., Biswas M., Bradley P., Bork P., Bucher P. (2002). InterPro: An integrated documentation resource for protein families, domains and functional sites. Brief Bioinform..

[29] Szklarczyk D., Kirsch R., Koutrouli M., Nastou K., Mehryary F., Hachilif R., Gable A.L., Fang T., Doncheva N.T., Pyysalo S. (2023). The STRING database in 2023: Protein–protein association networks and functional enrichment analyses for any sequenced genome of interest. Nucleic Acids Res..

[30] Metzger M.B., Hristova V.A., Weissman A.M. (2012). HECT and RING finger families of E3 ubiquitin ligases at a glance. J. Cell Sci..

[31] Liao Z., Chen X., Nie D., Wang J., Wu M. (2014). A RING finger protein 114 (RNF114) homolog from Chinese sturgeon (Acipenser sinensis) possesses immune-regulation properties via modulating RIG-I signaling pathway-mediated interferon expression. Fish Shellfish. Immunol..

[32] Rodriguez M.S., Egaña I., Lopitz-Otsoa F., Aillet F., Lopez-Mato M.P., Dorronsoro A., Lobato-Gil S., Sutherland J.D., Barrio R., Trigueros C. (2014). The RING ubiquitin E3 RNF114 interacts with A20 and modulates NF-κB activity and T-cell activation. Cell Death Dis..

[33] Shembade N., Ma A., Harhaj E.W. (2010). Inhibition of NF-κB Signaling by A20 Through Disruption of Ubiquitin Enzyme Complexes. Science.

[34] Maroof A., Patel D.D. (2018). TNF-α-induced protein 3 (A20): The immunological rheostat. J. Allergy Clin. Immunol..

